# Supplementary material for: Detergent selection as a determinant of protein melting behavior and drug-target interaction-calling in thermal stability proteomics
Source: bioRxiv. 2026 Jul 24:2026.05.28.728510. Preprint. [Version 2] doi: 10.64898/2026.05.28.728510 (PMC13419837; doi:10.64898/2026.05.28.728510)
Supplement: Supplementary file 1 [file NIHPP2026.05.28.728510v2-supplement-1.pdf]

bioRxiv preprint doi: https://doi.org/10.64898/2026.05.28.728510; this version posted July 24, 2026. The copyright holder for this preprint (which was not certified by peer review) is the author/funder, who has granted bioRxiv a license to display the preprint in perpetuity. It is made available under a CC-BY 4.0 International license.

## *Supplementary Information*

**Supplemental Figure 1.** Four-parameter fit of melting curves identifies outlier melting behavior.
**Supplemental Figure 2.** Relative qualitative behaviors of various detergents in melting buffers for thermal profiling.
**Supplemental Figure 3.** Different metrics to evaluate melting profiles of proteins from isothermal melts.
**Supplemental Figure 4.** Melting profile bar graphs for selected proteins from pfam and Jensen's compartments terms.
**Supplemental Figure 5.** Four-parameter melting curve fit to Reinhard, 2015 datasets for TPP in PBS or 0.4% NP-40.
**Supplemental Figure 7.** Western blot validation of TMEM97.
